# Seasonal Variation in the Voluntary Food Intake of Domesticated Cats (*Felis Catus*)

**DOI:** 10.1371/journal.pone.0096071

**Published:** 2014-04-23

**Authors:** Samuel Serisier, Alexandre Feugier, Sébastien Delmotte, Vincent Biourge, Alexander James German

**Affiliations:** 1 Royal Canin Research Center, BP 4, Aimargues, France; 2 MAD-Environnement Ltd, Nailloux, France; 3 Department of Obesity and Endocrinology, University of Liverpool, Leahurst Campus, Neston, United Kingdom; Université Catholique de Louvain, Belgium

## Abstract

There are numerous reports about seasonal cycles on food intake in animals but information is limited in dogs and cats. A 4-year prospective, observational, cohort study was conducted to assess differences in food intake in 38 *ad-libitum*-fed adult colony cats, of various breeds, ages and genders. Individual food intake was recorded on a daily basis, and the mean daily intake for each calendar month was calculated. These data were compared with climatic data (temperature and daylight length) for the region in the South of France where the study was performed. Data were analysed using both conventional statistical methods and by modelling using artificial neural networks (ANN). Irrespective of year, an effect of month was evident on food intake (*P*<0.001), with three periods of broadly differing intake. Food intake was least in the summer months (e.g. June, to August), and greatest during the months of late autumn and winter (e.g. October to February), with intermediate intake in the spring (e.g. March to May) and early autumn (e.g. September). A seasonal effect on bodyweight was not recorded. Periods of peak and trough food intake coincided with peaks and troughs in both temperature and daylight length. In conclusion, average food intake in summer is approximately 15% less than food intake during the winter months, and is likely to be due to the effects of outside temperatures and differences in daylight length. This seasonal effect in food intake should be properly considered when estimating daily maintenance energy requirements in cats.

## Introduction

In many mammalian species, the activity of physiological processes can vary throughout the year and, not uncommonly, alters with season. For example, in wild herbivores, a strong seasonal pattern exists for both reproductive and non-reproductive physiology [1], thought to be an evolutionary adaptation to ensure that reproduction and growth coincide with food availability. Cyclical annual changes in physiology are also seen in horses, whereby reproductive activity coincides with periods of long day length [2]. Additionally, physiological changes unrelated to reproduction occur including altered weight, body condition score (BCS), and feeding behaviour [3]. In this respect, activity and non-foraging behaviours are less pronounced in winter months, most likely so as to conserve energy. In order to maintain body weight and BCS, time devoted to feeding behaviour is greater during the spring months. Cyclical annual changes in feeding behaviour are also observed in wild carnivorous and omnivorous species. For instance, in honey badgers, foraging yield declines and dietary diversity increases in winter months [4] whilst, in arctic foxes, food intake declines during winter because of reduced food availability [5], [6]. However, the latter pattern of food intake is most likely to be directly due to the decreased food abundance rather than being driven by underlying physiological needs.

A number of studies have also examined seasonal food intake in domesticated species. In lactating dairy cows, exposure to a long-day photoperiod increases both milk production and food consumption, compared with lactating cows exposed to a short-day photoperiod [7], [8]. This effect is thought to be due to increased circulating insulin-like growth factor 1 concentrations in response to the longer day length [9]. In contrast, dry cows exposed to a long-day photoperiod consume less food than dry cows exposed to a short-day photoperiod [10]. The latter is thought to result from changes in feeding pattern caused by differences in light exposure: cows exposed to long days feed more directly after the food is presented, and less at other times; in contrast, cows exposed to a short-day photoperiod distribute their feeding throughout the day, leading to greater overall consumption [11]. The more complicated physiological changes seen in domesticated species likely result from variable husbandry, and the fact that food abundance is no longer a limiting factor. Studies conducted in the Antarctic have also identified adaptive changes in human physiology, whereby body weight and body fat mass increase during winter [12], and food intake also increases [13]. However, limited data are available from domestic pets, including cats. A recent study, from a temperate region of New Zealand, suggested that voluntary food intake in colony cats was influenced both by age and season [14]. Dietary energy intake was similar in young cats whatever the season, whilst intake was less in older cats during winter than summer [14]. Such observations were thought to be the result of increased growth rate of hair and increase in physical activity during the summer months, although neither was assessed during the study. In a second recent study, the effect of short-versus long-day photoperiod on food intake and physical activity was examined in indoor colony cats [15]. Compared with the short-day photoperiod, the long-day photoperiod increased physical activity and food intake. Whilst both of these studies might suggest that, in colony cats, greater food intake occurs during short photoperiods and/or winter months, neither examined possible seasonal effects throughout the year.

In recent years, various mathematical techniques have been developed to predict the behaviour of physiological processes from large datasets collected from living organisms. In biomedical research, such *in silico* experiments are beneficial in that they reduce reliance on *in vivo* experimentation and decrease experimentation costs [16]. This is because assumptions can be tested and data generated that would otherwise be difficult to measure. Among the various techniques used, artificial neural networks (ANN) are particularly recognised for their ability to predict very complex and non-linear processes, coupled with a certain ease and flexibility of implementation [17].

The authors recently reported a long-term observational study assessing food intake and body weight in a cohort of cats housed in a colony until 8 years of age [18]. In this study, a proportion of the cats gradually gained weight and had become overweight by 8 years of age. Importantly, a faster rate of growth in early life (<1 year of age) was the major risk factor predicting the likelihood of becoming overweight during adulthood. The aims of the current study were to use data from this same research colony, firstly, to clarify whether seasonal differences of food intake exist in cats maintained under domesticated conditions and, if so, to determine the influence of climatic conditions including ambient temperature and daylight length. A final aim was to use ANN to predict temporal variations of food intake from the existing physiological and environmental data, in order to improve understanding of its regulation.

## Materials and Methods

### Study Design

This was a prospective, observational, cohort study that assessed differences in voluntary food intake on a monthly and seasonal basis in a colony of domesticated cats. The study was conducted during the period of January 2006 to December 2009. This group of cats had previously been included in a study examining factors influencing body weight [18], although seasonal influences were not examined.

### Animals

Thirty-eight cats were included, 17 of which were male (15 neutered) and the remaining 21 female (10 neutered). Six were European shorthair, and the remaining were purebreds (including Abyssinian {2}, Bengal {7}, Birman {3}, Chinchilla {1}, exotic shorthair {3}, Maine coon {5}, Norwegian forest cat {1}, oriental {1}, Persian {3}, and Somali {2}). All cats were born in 2001 or 2002 and, as a result, median age, at study end, was 8.2y (range 7.4–9.6y). Twenty-two cats were in ideal condition (BCS = 5/9) [19], and the remaining 16 cats were overweight (BCS >5/9; BCS 6/9 [n = 6], BCS 7/9 [n = 5], BCS 9/9 [n = 5]). No major illnesses occurred in any of the cats during the course of the study.

### Housing and Husbandry

The study was performed at the Royal Canin Research Center, Aimargues, France. This site is located in the south of France where there is a Mediterranean climate, with mild, somewhat wet winters, and very warm, rather dry summers. Housing and treatment protocols adhered to European regulatory rules for animal welfare; all experimental protocols complied with European Union guidelines on animal welfare and were approved by the Royal Canin committee for animal ethics and welfare. Cats were housed in closed indoor-outdoor runs with 30 having unlimited outdoor access, and the remaining 8 housed exclusively indoor. The size of all runs was 27 m^2^, and there were a maximum of 8 cats per run. The runs with outdoor access were divided into an indoor part (of 13 m^2^) and an outdoor part (of 14 m^2^). Dependent on the season, the inside, temperature varied between 18°C and 24°C. Artificial light was provided in addition to the natural light, between 07.30 and 17.00, if natural light was judged to be insufficient by the animal caregivers. The decision to provide extra light was based upon the subjective impression of light intensity after a visual inspection of the facilities. For all cats, caregivers stimulated play behaviour for approximately 2 h, per run, per day. All cats remained healthy for the duration of the studies.

### Feeding Regime

The cats involved were used in feeding performance studies during the period. For 85% of the time, the whole group of cats were fed the same basal diet *ad libitum* (Table 1). The rest of the time (15%) was devoted to the feeding studies themselves, which occurred throughout the year, with no seasonal pattern. During each study, the whole group would be offered two diets *ad libitum*. The whole group was always fed the same two foods, but the exact diets offered would be changed on a daily basis (depending upon which diets were being tested at the time). All were dry expanded diets, which were complete and balanced. The variation in the overall dietary composition and metabolisable energy amongst diets is given in Table 2. Each cat had access to its own food station by microchip recognition, individual food intake (FI) was recorded daily using electronic weigh scales (M-Tronic Paris; France; accurate to within 0.5 g), and the mean food intake (in grams) was then automatically calculated. Body weight was also recorded, on a monthly basis, using the same calibrated electronic weigh scale (SG16000; Mettler Toledo, Albstadt, Germany; accurate to within 1 g).

**Table 1:** Dietary composition of the basal diet used for the study cats.

Criterion	Diet composition		
ME content^1^	16161kJ/kg (3860 kcal/kg)		
	Per 100g AF	g/1000 kcal (ME)	g/MJ (ME)
Moisture	7	18	4
Crude protein	32	83	20
Crude fat	15	39	9
Crude fibre	5.5	14	3
Total dietary fibre	11	28	7
Ash	6.8	18	4
Nitrogen free extract	33.7	87	21
Essential amino acids			
Arginine	1.9	4.9	1.2
Histidine	0.6	1.6	0.4
Hydroxyproline	0.9	2.2	0.5
Isoleucine	1.1	2.9	0.7
Leucine	2.7	6.9	1.6
Lysine	1.5	3.9	0.9
Methionine	1.1	3.0	0.7
Methionine and cystine	1.5	4.0	1.0
Phenylalanine	1.3	3.3	0.8
Taurine	0.2	0.5	0.1
Threonine	1.1	3.0	0.7
Tryptophan	0.3	0.7	0.2
Tyrosine	1.0	2.6	0.6
Valine	1.4	3.6	0.9

AF, as fed; ME, metabolisable energy. ^1^Measured in animal trials according to the 2010 American Association of Feed Control Officials (AAFCO) protocols [31] [30].

**Table 2:** Dietary composition of 15% remaining diets used in palatability trials in the study cats.

Criterion	Diet composition		
ME content^1^	16281 (12916–18597) kJ/kg (3895 [3090–4449] kcal/kg)		
	Per 100g AF	g/1000 kcal (ME)	g/MJ (ME)
Moisture	5.5 (5.5–8)	14 (12–23)	3 (3–5)
Crude protein	34 (23–46)	84 (61–127)	20 (15–30)
Crude fat	15 (9–25)	39 (28–56)	9 (7–13)
Crude fibre	4.3 (1.3–14.1)	11 (3–46)	3 (1–11)
Total dietary fibre	11 (6.1–23)	28 (14–74)	7 (3–18)
Ash	7.4 (5.2–9.4)	19 (13–27)	5 (3–7)
Nitrogen free extract	33.4 (24.8–44.4)	88 (58–118)	21 (14–28)
Essential amino acids			
Arginine	1.8 (1.2–2.7)	4.6 (3.1–6.9)	1.1 (0.7–1.7)
Histidine	0.7 (0.5–1.1)	1.8 (1.2–2.8)	0.4 (0.3–0.7)
Hydroxyproline	0.5 (0.0–1.0)	2.4 (0.0–2.6)	0.3 (0.0–0.6)
Isoleucine	1.3 (0.8–1.8)	3.3 (2.0–4.7)	0.8 (0.5–1.1)
Leucine	2.6 (1.7–4.1)	6.7 (4.5–10.5)	1.6 (1.1–2.5)
Lysine	1.5 (0.9–2.7)	4.0 (2.4–6.9)	0.9 (0.6–1.6)
Methionine	1.0 (0.6–1.4)	2.5 (1.6–3.6)	0.6 (0.4–0.9)
Methionine and cystine	1.5 (1.1–2.2)	3.9 (2.7–5.5)	0.9 (0.7–1.3)
Phenylalanine	1.4 (0.9–2.1)	3.6 (2.3–5.4)	0.9 (0.5–1.3)
Taurine	0.2 (0.2–0.4)	0.6 (0.5–1.1)	0.1 (0.1–0.3)
Threonine	1.1 (0.7–1.6)	2.9 (1.9–4.0)	0.7 (0.5–1.0)
Tryptophan	0.3 (0.2–0.5)	0.8 (0.5–1.3)	0.2 (0.1–0.3)
Tyrosine	1.2 (0.7–1.6)	3.0 (1.9–4.2)	0.7 (0.5–1.0)
Valine	1.5 (1.0–2.0)	3.8 (2.6–5.2)	0.9 (0.6–1.2)

Results are expressed as median (range). AF = as fed; ME = ^1^Measured in animal trials according to the 2010 American Association of Feed Control Officials (AAFCO) protocols [31] [30].

### Daylight Length and Average Daily Temperature

Data on temperature and daylight length, for the duration of the study period, were acquired from the French National Meteorological Service (Météo-France, Saint-Mandé, France).

### Data Handling and Statistical Analysis

All study data were recorded in a computer spreadsheet (see Spreadsheet S1). Statistical analysis was performed using computer software (SAS 9 for Windows, SAS Institute Inc.), and significance was set at *P*<0.05 for two-sided analyses. Given that the residuals of each statistical model were not normally distributed, quantitative variables were ranked to perform non-parametric analyses. In this respect, analysis of variance or covariance were undertaken using linear mixed models. These models included fixed effects and the cat as a random term, taking into account that measures are repeated for each cat throughout the study. Two outcome variables were investigated, namely body weight (in kg) and food intake (in g/day). The body weight model included the following fixed effects: year, month, weight status (overweight [BCS 6–9/9] or ideal weight [BCS 5/9]), sex, neuter status (neutered or sexually intact), housing type (indoor only vs. indoor-outdoor), and the interactions year×weight status, year×month, month×weight status, year×month×weight status. With regard to food intake, the same fixed effects were investigated as for body weight, along with the additional covariate body weight at 2006.

To examine further the effect of month on food intake, the variables daylight length (in seconds [s] per day) and daily mean temperature (in degrees Celsius [C°]) were investigated. Since the correlation between daily temperature mean and daylight length was significant, these two variables were merged in a last covariate ‘daily temperature×daylight length’, for the final linear mixed model on food intake to replace the previous “month” factor. Results are expressed as median (range).

### Modelling using Artificial Neural Networks (ANN)

All the calculations were carried out using R software (v 2.15.2) with the NNET package dedicated to ANN modelling. In order to understand better the mechanisms responsible for regulating food intake, ANNs were used [20] [21]. A multi-layer feed-forward neural network, so-called Multi-Layers Perceptron (MLP) was used, incorporating a back-propagation algorithm as previously described [22], [23], [24]. MLP is used to predict the mean value of a dependent variable conditionally to a vector of values of a set of predictive variables. In this approach, the network constructs a model based on examples of data with known outputs. The model is built solely from the examples presented, which are assumed to contain the information necessary to establish the relation.

For the MLP of the current study, a coherent scale between the different variables was ensured, by normalising the input and the output variables within a uniform range of 0 to 1 using the following equation:
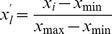
Where 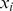 is the i^th^ value of a variable *x*, 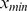 is the minimal value of the variable *x*, 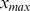 is the maximal value of the variable *x*, and predicted values can be de-normalised at the output of the ANN.

Next, the data were divided into two subsets: one was used in the training phase, and comprised 60% of the initial dataset; the other was used for the validation phase, and comprised the remaining 40%. To facilitate this, 29 of the 48 months of the study, with 38 cats, were randomly sampled for the training phase and 19 months with the same cats used for the validation phase. In order to build confidence intervals for the predictions, a bootstrap process was applied [25]: the sampling process previously described was repeated 1000 times, and the training-validation process was applied each time.

The performance of each MLP generated was measured, by calculating both the training mean squared error (MSEt) and the validation mean squared error (MSEv). To this end, the lower MSEv, the better the model. The average performance and the best performance were calculated. For each individual, a mean food intake prediction was calculated on the 1000 simulations with its standard deviation. MLPs were used to predict all the individual data (both used in training and validation phases), and the predictions were averaged by date. Coefficients of determination R^2^ were then calculated between measured and predicted mean values of food intake.

## Results

### Food Intake

**Figure 1:**
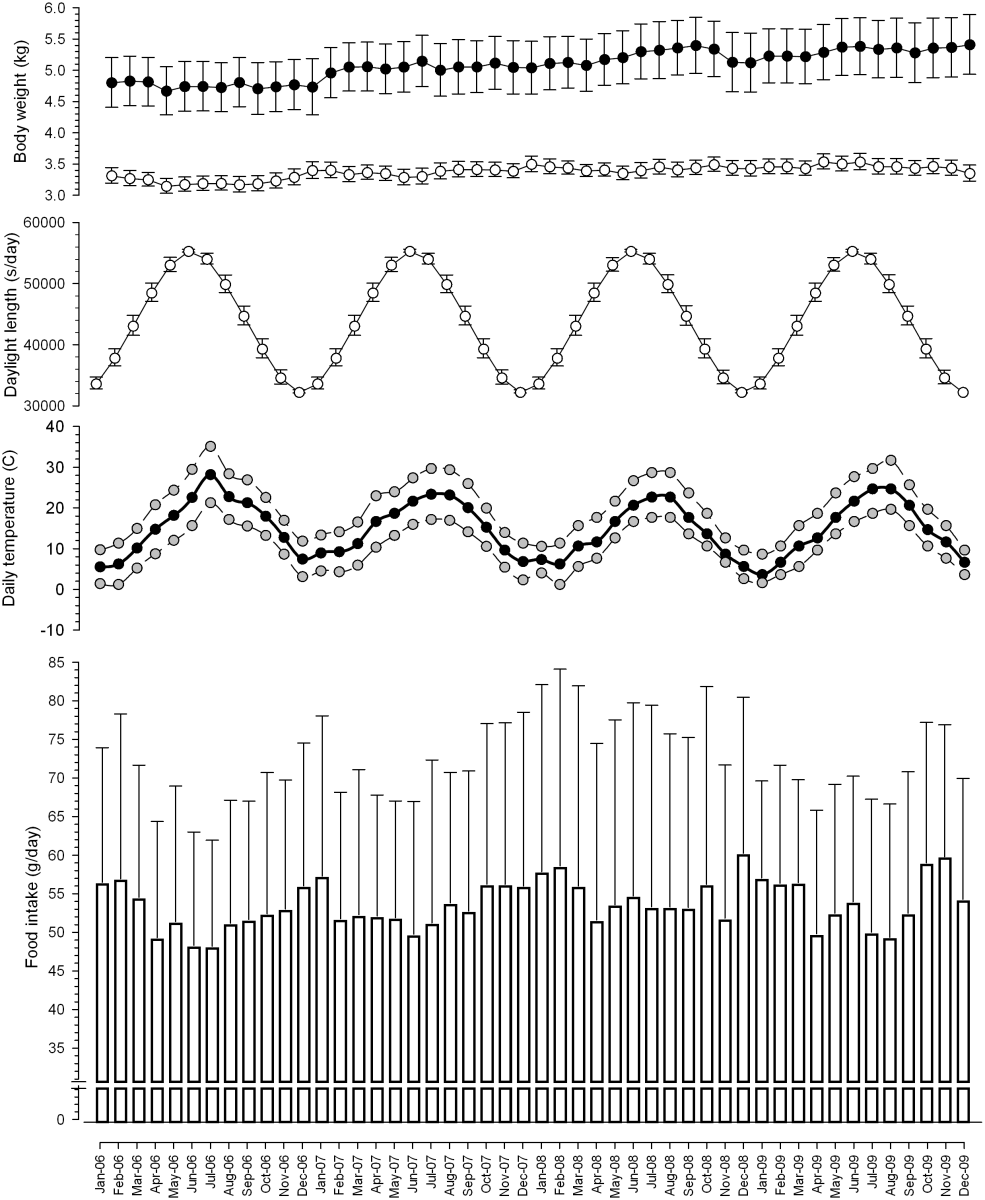
Environmental (e.g. ambient temperature and daylight length) and physiological (e.g. body weight and food intake from the study cats) parameters during the course of the study. Bodyweight (in kg) is expressed as median (black circles) and range (error bars) kg); daylight length (in min/day) is expressed as mean (open circles) and standard error (error bars) for each month; daily temperature (in Celsius) is expressed as mean (solid line and black circles) and minimum and maximum (dashed lines and open circles above and below the mean line); and food intake (in grams/day) is expressed as mean (white columns) and standard deviation (error bars) intake. Daylight length, temperature and food intake showed a seasonal pattern (*P*<0.001 for all). In contrast no seasonal pattern was observed for body weight, which increased steadily during the course of the study.

Food intake was assessed in 38 cats over a period of four years. Given that, at the outset, overweight cats were heavier than those in ideal weight (*P*<0.001), there were concerns that this might influence results. To account for this, statistical analyses included mean bodyweight in 2006 as a covariate. There was a significant effect of month on food intake (*P*<0.001), with a cyclical pattern, comprising three periods of broadly differing intake (Figure 1). Food intake was least in the summer months (e.g. mean ± standard error food intake day: June 52±2.0 g, July 51±2.3 g, August 52±2.2 g), and greatest during the months of late autumn and winter (e.g. October 56±2.3 g, November 55±2.2 g, December 57±1.9 g, January 57±2.2 g and February 56±2.3 g). For the months of spring and early autumn, food intake was intermediate between annual peak and trough intake (e.g. March 55±2.2 g, April 51±2.1 g, May 52±2.1 g, September 53±2.1 g). No effects of sex (*P* = 0.71), neuter status (*P* = 0.70), weight status (*P* = 0.45), or housing type (*P* = 0.41) were seen. However, similar to a previous study [18], which included these cats within a larger group, there was a significant effect of year of study on food intake, with a significant interaction between year and weight status (*P*<0.001). In this respect, food intake was greater in 2008 compared with 2009 in overweight cats only (*P* = 0.02). However, no interaction was seen between weight status and month (*P* = 0.11) and between weight status, month and year (*P* = 0.98). The reason for the difference in food intake between years in overweight cats is not known, but this was unrelated to the observed seasonal differences, given the lack of association with month effect.

### Body Weight

The effect of season on bodyweight was also assessed and, in contrast to food intake, no month effect was seen (*P* = 0.96). Instead, there were significant effects of year (e.g. 2006: 3.65 kg [2.06–9.07 kg]; 2007: 3.75 kg [2.12–9.55 kg]; 2008: 3.82 kg [2.17–10.05 kg]; 2009: 3.82 kg [2.19–10.20 kg]; *P*<0.001; Figure 1), sex (*P* = 0.006), and weight status (*P*<0.001), but not neuter status (*P* = 0.94), or housing type (*P* = 0.69). Further, there was also a significant interaction between weight status and year (*P* = 0.03): in this respect, the body weight of overweight cats increased steadily over the course of the study (weight at start 4.80±0.40 kg vs. weight at end 5.41±0.48 kg), whereas the bodyweight of ideal weight remained stable (weight at start 3.32±0.1 kg vs. weight at end 3.36±0.13 kg). The reason for these changes has been discussed in a previous publication in which these cats were included [18].

### Effects of Average Temperature and Daylight Length on Food Intake

Data on average temperature and daylight length were examined, for the duration of the study period. As with food intake, both mean daily temperature and mean monthly daylight length were cyclical, with peaks and troughs in both temperature (peak monthly temperature in July, 25.1±2.4°C; trough monthly temperature in January, 6.7±2.3°C) and daylight length (longest total daylight length July, 54103±891 sec; shortest total daylight length, January 33751±960 sec) occurring at similar times to food intake.

The effects of both temperature and daylight length on food intake were then assessed. Non-parametric ANCOVA was repeated including mean monthly temperature and daylight length, as covariates, instead of month. Once again, there were effects of year of study (*P* = 0.02) and mean body weight in 2006 (*P*<0.001) on food intake, but no effect of sex (*P* = 0.57), neuter status (*P* = 0.61), weight status (*P* = 0.61), or housing type (*P* = 0.40). Further, there was a significant effect of daylight length (*P* = 0.016), but not temperature (*P* = 0.28). However, given that perfect correlation existed between these two variables (Kendall’s tau = 1.00, *P*<0.001), it was difficult to dissociate these two effects with certainty. Therefore, a new parameter (temperature×daylight length) was then created to assess the combined effect of these variables. There were effects of mean body weight in 2006 (*P*<0.001), temperature×daylight length (*P*<0.001), and year of study (*P* = 0.049), but not sex (*P* = 0.57), neuter status (*P* = 0.61) or weight status (*P* = 0.51), on food intake.

### Modelling

**Figure 2:**
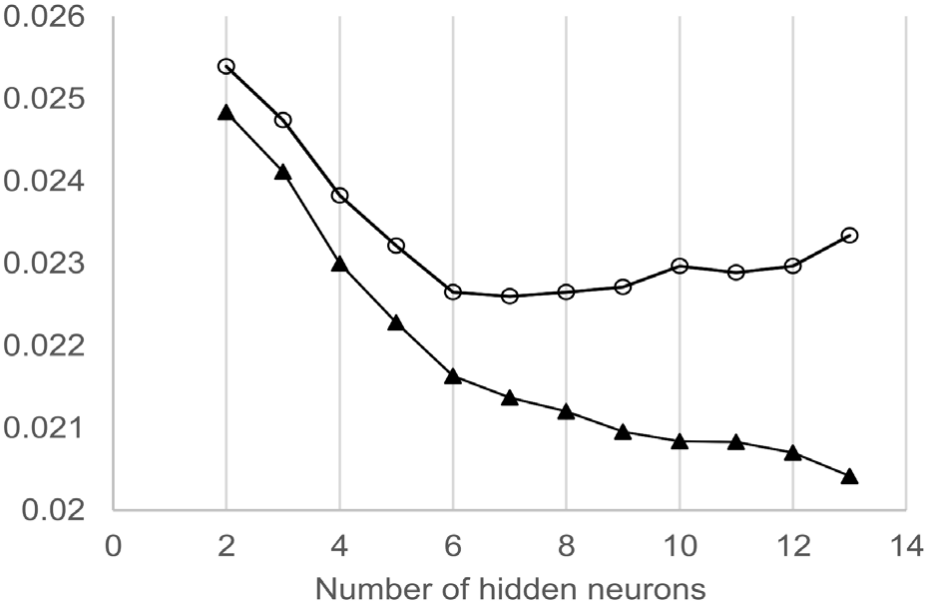
Determination of the best multi-layers perceptron (MLP) architecture (i.e optimal number of hidden neurons). The curves show mean squared errors in function of the number of hidden neurons for the training (MSEt, black triangles) and the validation phase (MSEv, open circles) of the modelling process. MSEt and MSEv were averaged over the 1000 simulations carried at each level of hidden neurons, with the following predictive variables: year, temperature, daylight length, temperature x daylight length, body weight. As the number of neurons increased, training performance improved (decreasing MSEt), whilst validation performance firstly improved (decreasing MSEv) but then worsened (MSEv increasing) at 8 hidden neurons. Thus, the optimal state was reached at 7 neurons.

The input variables chosen in modelling were determined by the results of the previous statistical analyses. Retained predictive variables were: year, temperature, daylight length and initial body weight. Additional tests were conducted using sexual status or month of the year (as a numerical value), and confirmed that they were not relevant in the model. Different combinations of the chosen variables were used to determine simultaneously the best MLP architecture (i.e. optimal number of hidden neurons) and the best set of predictive variables. Figure 2 illustrates the process of determining the optimal number of hidden neurons: as the number of neurons was increased, the training performance improved (decreasing MSEt) whilst, simultaneously, the validation performance firstly improved (decreasing MSEv) but then worsened (MSEv increasing) at 8 hidden neurons. Thus, the optimal state was reached at 7 neurons.

**Figure 3:**
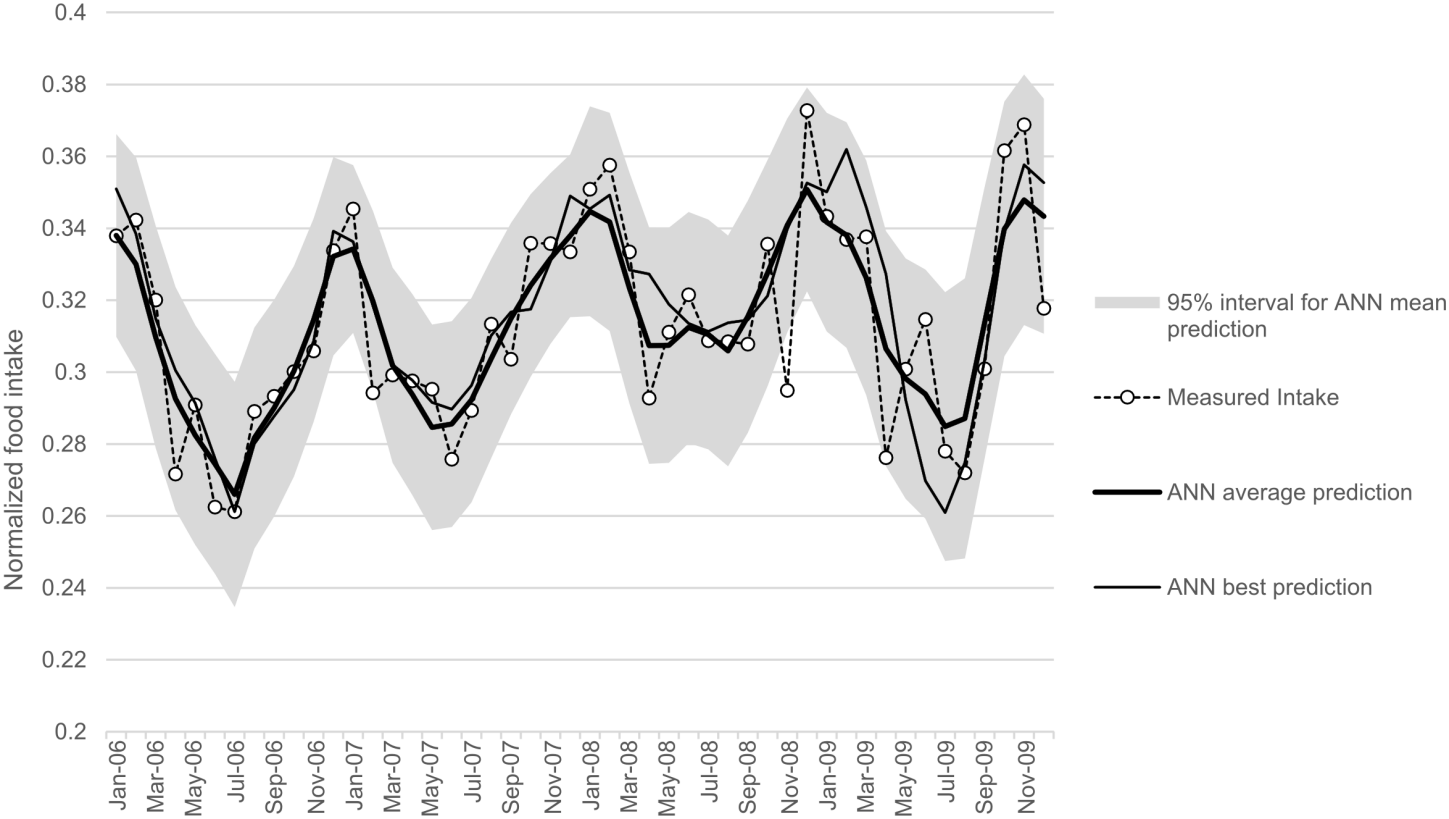
Measured and predicted average food intake, over the 4-year study by using artificial neural network (ANN) so-called multi-layers perceptron (MLP). Open circles and dashed lines represent the measured intake. The thick solid line represents the MLP average, the solid thin line represents the best MLP, Finally, the area shaded in grey represents the 95% CI for the MLP prediction. As the number of neurons was increased, the training performance improved (decreasing MSEt). The best input variables to predict food intake were year, temperature, daylight length, temperature×daylight length and body weight.

The results of the modelling processes are shown in Table 3. Three combinations of input variables were tested, giving close MSEv values, with R^2^ varying between 0.59 and 0.75. Thus, the input variables that best predicted food intake were year, temperature, daylight length, temperature×daylight length and body weight. Figure 3 shows the temporal evolution of measures and predictions of the average food intake, with predictions based on the results of the best MLP. An association was identified between cyclical variations in food intake, and the cyclical environmental variations (i.e. daylight length and temperature).

**Table 3:** Comparison of input variables combinations to predict food intake using artificial neural networks.

Predictive variables	ANN MLP			
	Optimal number of neurons	MSEt	MSEv	R^2^
Year, Temperature, Daylight length, Body weight	8	0.0213	0.0231	0.7
Year, Temperature x Daylight length, Body weight	8	0.0214	0.0236	0.59
Year, Temperature, Daylight length, Temperature x Daylight length, Body weight	7	0.0212	0.0226	0.75

ANN MLP, Artificial neural network multi-layers perceptron; MSEt, training mean square error; MSEv, validation mean square error; R^2^, coefficient of determination.

## Discussion

The results of the current study have demonstrated that the voluntary intake of domestic cats, fed *ad libitum*, follows a cyclical pattern, being greatest in late autumn to winter, and least in summer. The fact that these changes did not lead to significant changes in body weight suggest that they occurred in response to changes in energy needs, for instance due to changes in energy for thermoregulation or activity. These results are of importance to feline nutrition, most notably to domesticated cats, and suggest feeding strategies might need to be adapted on a seasonal basis to ensure that availability meets demands throughout the year. Nonetheless, given that the cats were maintained in a colony, the results are not necessarily applicable to cats housed in other settings, or indeed wild felids. As part of the current study, both traditional statistical methods and ANN were also used to determine the factors responsible for differences in food intake, and most important were ambient temperature, daylight length, or a combination of the two. A number of possible explanations exist for the results observed. First, the seasonal changes observed in food intake could be the result of differences in ambient temperature, whereby more energy is required for thermogenesis during late autumn and winter, than in summer. Alternatively, the key trigger for the food intake differences could be daylight length. Given that ambient temperature and daylight length were closely correlated with one another, it was not possible to separate their individual effects on food intake, despite advanced modelling techniques. In fact, the effects of temperature and daylight length may not be mutually exclusive; for instance, it is feasible that changes in daylight length might be the stimulus to trigger changes in food intake and prepare the cat for increased thermogenesis. Further studies would be needed to separate these effects, for example by exposing cats to long and short photoperiods during summer and winter.

Another possible explanation for the observed differences in food intake would be differences in activity levels, which itself might not be independent of ambient temperature or daylight length. Indeed, the increased heat production required to maintain body temperature during colder periods might result from increased physical activity. All cats were given an opportunity to exercise, which included voluntary periods of activity whilst outside and in the runs, and also play sessions whereby caregivers encouraged movement. The housing conditions, periods available for free activity, and periods of play activity did not vary during the course of the study and definitely did not vary throughout the year. Thus, it is unlikely that differences in activity would be the driving force for the cyclical differences in food intake observed. However, because activity was not measured objectively in the study, for instance using accelerometers, we cannot discount this possibility altogether.

In a previous study, which included data from the current study cats, long-term changes in food intake and bodyweight were also assessed when fed a dry extruded diet *ad libitum* [18]. Two phenotypes of cats were identified: cats with the ‘ideal weight’ phenotype regulated their food intake and bodyweight throughout life, despite *ad libitum* access to food; in contrast, cats with the ‘overweight’ phenotype grew rapidly, and were already heavier at 12 months of age, then continued to gain weight progressively thereafter. Food intake was greater in the overweight phenotype cats throughout the course of the study. Although the reasons for these differences were not known, various possibilities were suggested, including genetic and epigenetic differences, *in utero* factors, difference in physical activity, differences in feeding-related behaviour, digestibility differences, and possible differences in gastrointestinal microbiota [18]. Not surprisingly, given that the current study cats were also part of the previous study, similar findings were again observed, and the reasons for these findings are likely to be the same. However, most fascinating is that, despite the fact that ‘overweight phenotype’ cats were unable to regulate their food intake to maintain body weight long term, they still demonstrated seasonality in their food intake. Thus, whilst physiological cues driving seasonal variation appear to function effectively, other physiological mechanisms governing food intake (e.g. appetite regulation) do not. Of course, it is unclear as to whether or not the same findings would be seen if a different feeding strategy were used. It would be fascinating to study feeding behaviour in feline species in the wild, most notably the wild progenitors of domestic cats such as the European wildcat, near Eastern wildcat, and the central Asian wildcat [26]. Since such species are not prone to becoming overweight, it would perhaps be the best setting to examine the seasonality effects in isolation.

The findings of the current study contradict the findings of a previous study in cats where food intake was compared between study periods in winter and summer [14]. In that study, dietary energy intake was similar in young cats whatever the season, but less in older cats during winter than in summer. The reason for this is not clear, but might be the result of differences in the populations studied and methodology used. In the previous work, cats were fed to maintain body weight, whilst cats were fed *ad libitum* for the current research; this is likely to have lead to differences in physiological responses to feeding. Further, in the previous study, food intake was only assessed during 2 four-week test periods in summer and winter, whilst it was continuously monitored over a four-year period in the current study. The population in the previous study was smaller, all cats were domestic shorthair and neutered, and separate groups of young and old cats were used. In contrast, the current study population was larger, both neutered and entire cats were included and there was a range of breeds. Further, all cats were approximately the same age, although the fact that intake was assessed over four years did enable age to be considered. There were differences in housing, with the current study cats being housed either exclusively indoors or having some access to the outdoors, whilst groups were either exclusively indoors or exclusively outdoors in the previous work. Other possibilities include differences in climate between the locations, with the previous study being conducted in a temperate region of the southern hemisphere, whilst the current study was conducted in a Mediterranean climate. As a result, it would be worth considering further studies where the methodology of these two studies is combined, for instance by comparing *ad libitum* food intake versus feeding to maintain weight, in different ages, throughout a 12-month period.

The current results also contrast with a second recent study, which demonstrated greater physical activity and energy intake during a long-day (16 h light: 8 h dark) photoperiod compared with a short-day (8 h light: 16 h dark) period [15]. Again, methodological differences might explain why these results conflict with the results of the current study. First, cats in the previous study were exclusively housed indoors in artificial light, and ambient temperature was closely controlled (e.g. between 20–22°C). In contrast, most cats in the current study had access to outdoors, were exposed to natural light (with supplemental artificial light supplied if required), and the ambient temperature was less closely controlled (e.g. between 18–24°C indoors, with much greater temperature variability when cats were outside). Thus, the previous study arguably only assessed differences between photoperiod extremes at the same ambient temperature, whilst the current study assessed the complete spectrum of seasonal changes in both light and temperature. Second, cats in the current study were fed *ad libitum*, whilst food intake of the cats in the previous study was regulated to maintain body weight. As a result, the differences in food intake in the previous study were likely to be secondary to differences in physical activity, as suggested by the study results, whereas the other factors might have affected food intake in the current study. For example, there would likely have been a greater need for the cats of the current study to thermoregulate when outdoor temperatures were low in winter, whilst the extremely warm outdoor temperatures during the height of the Mediterranean summer might have markedly suppressed physical activity at this time. Physical activity, metabolic rate and body composition were assessed in the previous study, but were not assessed in the cats of the current study. For future studies, it would be worth measuring seasonal changes in all such parameters in addition to food intake and body weight.

In some other species, increased food intake occurs at different times of the year than was observed in the cats of the current study. For instance, increased food intake is noted in spring in some wild carnivorous and omnivorous species [4]. However, in these cases, food intake may be responding to food source availability, which is scarce over the winter and relatively plentiful from spring to autumn. Further, in these wild species, weight loss often occurs during periods of limited food resource. Given that the cats in the current study were fed *ad libitum*, and weight loss was not observed, food availability was not a limiting factor. Further, although day-to-day fluctuations might have been possible, the fact that monthly body weight measurements either remained stable or increased slightly over time, suggests that cats were in neutral to positive energy balance throughout the study, rather than facing cyclical periods of positive and negative energy intake. Thus, different physiological mechanisms are likely to account for the differences in food intake amongst these species and environments. In other species, food intake does not reflect availability of resource, but differences in behaviour of physiology. For instance, in some species food intake increases when mating behaviour is maximal. This would appear to be a logical response since a positive energy balance is likely to improve reproductive status and chance of success, both in successfully finding a mate, in successful conception, and in successful parturition, and subsequent lactation. An extreme example of food intake responding to needs of reproduction and lactation is the dairy cow, and it is noteworthy that the responses to food intake are different between lactation and the dry period. In the current study, the cats were not part of a breeding colony and, indeed, the majority were neutered reducing the influence of reproductive hormones on feeding behaviour. Thus, behavioural differences relating to reproductive status are unlikely to have markedly influenced food intake. Further, there were no statistical differences noted, either between sexes or cats with different neuter status. That said, only two entire male cats were included, which might have meant an effect were missed. Further, information on the timing of oestrus was not recorded; if it had been, it might have been possible to identify a minor effect of sex hormones on food intake in entire female cats. Finally, even despite neutering, there may still have been sex-related behavioural differences, if they did not involve reproductive hormones. Therefore, further work would be required to determine the true effects of sex and reproductive status on food intake in domesticated cats.

As previously reported in this population [18], a gradual increase in mean bodyweight was seen over the course of the 4-year period suggesting that, throughout the study, food intake marginally exceeded requirements. It is unclear as to what effect this positive energy balance might have had on the study results. In a previous study, a long photoperiod in obese Zucker rats resulted in greater body weight than those maintained in a short photoperiod, but such an effect is not seen in lean rats [27]. Thus, we cannot completely discount a confounding effect of this positive energy balance in the current study. That said, no effect of weight status (overweight vs. ideal weight) was observed suggesting that this was not a major confounding factor in the study.

In the current study, the process of random selection of input data during the training phase was repeated 1000 times, to minimise the possibility of learning being biased by a particular data set. The results reported represent the average prediction performed with the 1000 trained ANN, the prediction intervals calculated on these 1000 runs (each run is performed with a ANN trained with new set of data), and the best prediction ANN among these 1000 runs. Despite the limited number of predictive variables and the complexity of the modelled process, the MLP results can be considered to provide a good prediction of the mean food intakes. The fact that a small number of environmental variables could be used to predict average intake suggests that supervised ANNs are a good predictive tool for modelling such a complex physiological process. That said, as with any statistical modelling approach, the model output could change if new data were added to the dataset. Further, performance was not perfect (R^2^ results of 0.59–0.75) and a significant amount of variability in food intake remained unaccounted for. This variability is likely to be the result of other unmeasured variables, which might be cat-specific (e.g. underlying physiological conditions, taste preferences and feeding habits of the cat) or environmental (e.g. external noise levels). Unfortunately, these factors were not recorded in the current study. ANN modelling works well when the data set is large enough and is an accurate way of maximising the potential of such data in a predictive goal. ANNs are not designed to understand a process, but only to mimic its outcomes. Therefore, they are particularly useful in forecasting and in helping in decision-making in various fields [28], [29], [30]. To be a useful tool for predicting food intake, models should not be restricted to environmental factors because it limits the prediction ability to average food intakes, and does not integrate individual variability. A future aim would be to improve the food intake modelling in targeting individuals. This would require a coupling, in the same model, of individual descriptors of physiological conditions and environmental factors (including the factors studied and additional factors). Such a study would require a more complex design to account for the measurement of a wider range of individual and environmental factors.

This study has some limitations, the first of which was that exact metabolisable energy intake was not known, because such data were not available. Since, a range of diets were fed throughout the course of the study, it is possible that this might have influenced our results. Diets differed in a number of ways including energy content, ingredients, and macronutrient levels, and all such factors might feasibly have influenced food intake. However, the feeding studies were performed throughout the year, and there were with no seasonal differences in when these occurred. Therefore, whilst this is a potential confounding factor, it is unlikely to have had a systematic influence on the seasonal food intake of the cats in the current study. A second limitation was the fact that additional light was used to supplement natural light, if judged to be insufficient by the animal caregivers. Such decisions were subjective and, therefore, introduced variability in the exact amount of light available. This limitation is partly offset by the fact that the same people always made this decision. That said, for future studies, it would be worth standardising the delivery of artificial light, for instance by using a light meter to determine when it was required. A further issue with the use of supplemental light was the fact that the exact amount provided was not recorded, and might be a confounding factor. In this respect, more supplemental light is likely to have been given during periods of short natural daylight length than for periods of long natural daylight. Thus, the provision of supplemental light may have reduced the overall effect of daylight length on food intake. That said, this confounding effect is likely to have been relatively minor. In this respect, based upon the available meteorological data, the shortest recorded daylight length was 34260 s (or 9.0 h), and the longest daylight length was 55320 s (or 15.4 h). When supplemental artificial light was required, it was only provided between 07∶30 and 17∶00, a maximum period of 9.5 h. Thus, at most, the provision of supplemental light would only have lead to an additional 30 minutes of light on the shortest day. Despite this, the provision of supplemental light remains a limitation, and it would be preferable to record the exact amount provided in any future study.

A third limitation was that eight of the cats were exclusively housed indoors, meaning that the effects of outdoor temperature could have been less marked in this group. That said, no effect of housing conditions (indoor only versus indoor-outdoor) was seen on either food intake or body weight. A fourth limitation was the fact that we aimed to study domesticated cats but, arguably, the environment in which the cats were housed was not representative of cats living in a domestic environment. A future study could assess food intake in pet cats. However, such a study would be difficult to undertake in a field setting, since fewer factors can be controlled, and collection of data may be tricky. Most notably, it might be difficult to measure food intake accurately, and to ensure that other food sources are not available. There would also be great diversity in environment, most notably ambient temperature, access to outdoors, provision of artificial light and household temperature. Thus, a large population of pet cats would need to be studied to ensure that such increased variability did not mask any genuine effects.

In conclusion, a seasonal effect on voluntary food intake exists in domesticated cats, whereby food intake is greater in winter and less in summer. These changes in food intake are likely to be the result of changes in ambient temperature, daylight length, or both, and the fact that they do not cause bodyweight changes suggest that they occur in response to changes in energy requirements. The possible effect of climatic variation on voluntary food intake in pet cats should be considered in companion animals, and may mean that feeding strategies may need to be seasonally adjusted, to ensure that availability meets demand at different times of the year. Further studies are needed to understand the metabolic mechanisms involved in this seasonal effect, and to determine whether similar effects are seen in wild felids.

## Supporting Information

Spreadsheet S1 **Computer spreadsheet summarising all of the data used in the study.** Separate columns are include of cats identification number (cats), overweight status (group), year, month, food intake (in g), body weight (in kg), sex, sexual status (I = intact; N = neutered), average monthly temperature (°C), daylight length (in s), and the composite of temperature and daylight length (T°×Daylight).
(XLSX)
